# Identification of a Prognostic Model Based on Fatty Acid Metabolism-Related Genes of Head and Neck Squamous Cell Carcinoma

**DOI:** 10.3389/fgene.2022.888764

**Published:** 2022-06-30

**Authors:** Peiyu Du, Yue Chai, Shimin Zong, Jianxin Yue, Hongjun Xiao

**Affiliations:** ^1^ Department of Otorhinolaryngology, Union Hospital, Tongji Medical College, Huazhong University of Science and Technology, Wuhan, China; ^2^ Department of Medical Oncology, National Cancer Cente, Chinese Academy of Medical Sciences and Peking Union Medical College, Beijing, China

**Keywords:** fatty acid metabolism, head and neck squamous cell carcinoma, prognosis, risk signature, lasso-cox regression

## Abstract

The fatty acid metabolism (FAM) is known to impact tumorigenesis, tumor progression and treatment resistance via enhancing lipid synthesis, storage and catabolism. However, the role of FAM in head and neck squamous cell carcinoma (HNSCC) has remained elusive. In the present study, we obtained a total of 69 differentially expressed FAM-related genes between 502 HNSCC samples and 44 normal samples from The Cancer Genome Atlas (TCGA) database. The HNSCC samples were divided into 2 clusters according to 69 differentially expressed genes (DEGs) via cluster analysis. Then DEGs in the two clusters were found, and 137 prognostic DEGs were identified by univariate analysis. Subsequently, combined with the clinical information of 546 HNSCC patients from TCGA database, a 12-gene prognostic risk model was established (FEPHX3, SPINK7, FCRLA, MASP1, ZNF541, CD5, BEST2 and ZAP70 were down-regulation, ADPRHL1, DYNC1I1, KCNG1 and LINC00460 were up-regulation) using multivariate Cox regression and LASSO regression analysis. The risk scores of 546 HNSCC samples were calculated. According to the median risk score, 546 HNSCC patients were divided into the high- and low-risk (high- and low score) groups. The Kaplan-Meier survival analysis showed that the survival time of HNSCC patients was significantly shorter in the high-risk group than that in the low-risk group (*p* < 0.001). The same conclusion was obtained in the Gene Expression Omnibus (GEO) dataset. After that, the multivariate Cox regression analysis indicated that the risk score was an independent factor for patients with HNSCC in the TCGA cohort. In addition, single-sample gene set enrichment analysis (ssGSEA) indicated that the level of infiltrating immune cells was relatively low in the high-risk group compared with the low-risk group. **In summary**, FAM-related gene expression-based risk signature could predict the prognosis of HNSCC independently.

## 1 Introduction

Head and neck squamous cell carcinoma (HNSCC), the most common pathological type of head and neck cancer, arises from the mucosal epithelium in the oral cavity, pharynx, and larynx (Johnson et al., 2020). HNSCC is the 6th most common cancer worldwide (Sung et al., 2021). The onset of HNSCC is increasing with approximately 600,000 new cases are diagnosed each year (Solomon et al., 2018). Oralcavity and larynx cancers are often connected with factors such as smoking and alcohol abuse, or both, whereas pharynx cancers are attributable to human papillomavirus (HPV) infection, especially HPV-16. Therefore, HNSCC is divided into 2 groups: HPV positive HNSCC with better prognosis and HPV negative with worse prognosis (Johnson et al., 2020). Early-stage HNSCC is typically asymptomatic and most HNSCC is already locally advanced or advanced when diagnosed. As a result of no early effective detection test or screening method for HNSCC being available currently, a careful physical examination has far remained the primary diagnostic tool in early detection of HNSCC. At present, treatment approaches for HNSCC are mainly comprehensive modalities with surgery, radiotherapy, chemotherapy, targeted therapy, immunotherapies, et al. However, the overall responses rates of patients with HNSCC to targeted therapeutic drugs, such as epidermal growth factor receptor (EGFR) inhibitors (cetuximab) and programmed cell death protein 1 inhibitors (pembrolizumab and nivolumab), were moderate (Zhang et al., 2021). Thus, finding a novel prognostic biomarker for the treatment of HNSCC is a major goal. The fatty acid metabolism (FAM) occupies a key role in the entire lipid metabolism, which is known to impact tumorigenesis, tumor progression and treatment resistance via enhancing lipid synthesis, storage and catabolism (Fernández et al., 2020; Hoy et al., 2021). The biosynthesis of fatty acids (FAs) is activated in cancer cells to fulfill lipid synthesis of membranes and signaling molecules, and energy storage (Fhu and Ali, 2020). Compared with glucose oxidation, fatty acid (FA) oxidation is more likely to be utilized by tumor cells due to higher energy production (Li and Zhang, 2016). Increased FA oxidation confers survival advantages for tumors not only to resist chemotherapeutic and radiation treatments but also to alleviate cellular stresses involved in the metastatic cascade (Corn et al., 2020). In addition, FAM could have an impact on cellular phenotype and function of tumor-infiltrating immune cells in tumor microenvironments, which may be associated with immunosuppression (Corn et al., 2020). Deregulating or blocking FAs levels through the FAM-related pathway in cancer might inhibit tumor cell growth and therefore that identifying the targets to regulate FAM is essential. Several studies have shown that FAM was closely related to the oncogenesis and progression of breast cancer, colorectal cancer and liver cancer (Wang Y.-n. et al., 2018; Madak-Erdogan et al., 2019; He et al., 2021; Hofmanová et al., 2021). A previous study has demonstrated that the FA synthase inhibitor orlistat could reduce the growth and metastasis of orthotopic tongue oral squamous cell carcinomas (Agostini et al., 2014). Su et al. found that the overexpression of long-chain acyl-CoA dehydrogenase, a type of FA oxidation-related enzyme, had a protective effect on OS in advanced HNSCC(Su et al., 2020). To date, the role of FAM in head and neck squamous cell carcinoma has remained elusive.

In the present research, bioinformatics analysis was performed to investigate FAM-related genes that may play an important role in HNSCC and constructed a prognostic model for patients with HNSCC. The association between the expression of FAM-related genes and the prognosis of HNSCC was further analyzed via this model to explore the potential prognostic value of FAM-related genes and provide additional evidence for potential therapeutic targets for HNSCC.

## 2 Methods

### 2.1 Data Processing

Gene sets related to FAM were downloaded from the gene set enrichment analysis (GSEA, http://www.gsea-msigdb.org/gsea/msigdb/index.jsp) website. The RNA-sequencing (RNA-Seq) data of patients with HNSCC were searched in The Cancer Genome Atlas (TCGA) database (https://portal.gdc.cancer.gov/repository) and Gene Expression Omnibus (GEO) database (https://www.ncbi.nlm.nih.gov/geo). Their clinicopathological, genetic, epigenetic, and survival data were downloaded for secondary analysis.

### 2.2 Identification of Differentially Expressed Fatty Acid Metabolism-Related Genes

The R language “limma”package was used to detect differentially expressed genes (DEGs) between tumor and normal tissues of patients with HNSCC. A *p* value <0.05 was used as the screening criterion. HNSCC patients were clustered into two subgroups based on prognosis-based FAM-related genes which were identified by univariate Cox regression analysis using the R “**limma**” “survival” “**ConsensusClusterPlus**” packages. After that, survival analyses were performed in the 2 subgroups using the **“survival**” “**survminer**” packages in R. The *p* values <0.001 were considered statistically significant. DEGs between the two subgroups were analyzed by R “**limma**” package, quantified as the logFC >1 by R “**sva**” package, and plotted by R “**ggplot**” package. Heatmaps of these DEGs between the two subgroups were drawn with R “**pheatmap**” package.

### 2.3 Development and Validation of the Fatty Acid Metabolism-Based Prognostic Model

The samples from the TCGA database was acted as the training set to construct a prognostic model while the samples from the GEO database were designated as the test set to verify the accuracy of the prognostic model. The key prognosis-related FAMs were selected by the LASSO Cox regression analysis and multivariate Cox regression analysis via the R “**glmnet**” package. The risk score formula for the prediction of prognosis of patients with HNSCC was as follows: The risk score calculating formula is:

$$\mathrm{Risk}\;\mathrm{score}=\;\sum_{i=1}^{n}\mathrm{Coefi}*\mathrm{Expi},$$

where the n refers to the number of signature genes, Coefi refers to the coefficients, Expi refers to gene expression level. Then risk scores of HNSCC patients were obtained according to the risk model. Based on the median value of risk scores, HNSCC patients were divided into high- and low-risk subgroups. The R **“survival”** and **“survmine”** packages were used for survival analysis. Principal component analysis (PCA) and t-distributed stochastic neighbor embedding (t-SNE) were used for dimensionality reduction analysis via R “Rtsne” and “ggplot2” packages. The receiver operating characteristic (ROC) curve was drawn by R “timeROC” package to evaluate the accuracy of the risk model. The risk heat plot was plotted by the R “pheatmap” package. HNSCC cohort from the GEO database (GSE41613) was employed to validate the risk model. The differentially expressed FAM-related genes were obtained and the risk scores were calculated by the same methods used for the TCGA cohort. The patients with HNSCC in the cohort were also divided into high- or low-risk subgroups, and these subgroups were then compared to validate the risk model.

### 2.4 Human Protein Atlas

The protein expression levels of the hub genes were validated using the Human Protein Atlas (HPA) database (https://www. proteinatlas. org). HPA is a Swedish-based program initiated in 2003 for providing all human proteins in cells, tissues, and organs. Getting immunohistochemical data of patients with or without HNSCC from HPA helped us to further verify the protein expression levels of 12-genes that were identified in the risk model.

### 2.5 Independent Prognostic Analysis of the Risk Score

Univariate and multivariable Cox regression analyses including clinical characteristics and risk scores were performed to evaluate whether our ricsk score could serve as an independent prognostic factor for HNSCC and were visualized as forest plots and heatmaps using R “limma” and “pheatmap” packages. A *p* value <0.05 was considered significant.

### 2.6 Functional Enrichment Analysis

The DEGs of HNSCC patients in the high- and low-risk groups in the TCGA cohorts were calculated according to the risk model. Gene Ontology (GO) enrichment analysis of DEGs was performed to obtain the enrichment of DEGs regarding biological process, molecular function and cellular component, and Kyoto Encyclopedia of Genes and Genomes (KEGG) enrichment analysis was carried out to obtain signal pathways of DEGs using the R “clusterProfiler” and “enrichplot” packages (*p* value > 0.05 and q value < 0.05) (Ashburner et al., 2000; Kanehisa et al., 2017). The functional enrichment results of GO and KEGG analyses were visualized as bubble charts and barplots.

### 2.7 Estimation of Immune Status

Single-sample GSEA (ssGSEA) enrichment scores were calculated by the “GSVA” package of R to quantified the immune infiltration levels of the DEGs of HNSCC patients in the high- and low-risk groups in the TCGA cohorts. Patients were scored according to the risk model that was established in our study. The median value of the risk score was as a cut-off point. The high-risk group consisted of patients with risk scores above the median value, while the low-risk group consisted of patients with risk scores below the median value.

### 2.8 Statistical Analysis

Categorical variables between normal and HNSCC tissues were compared by the Pearson chi-square test. Survival analyses were performed by Kaplan-Meier Log-rank analyses. The differences in immune cell infiltration and immune pathway activation between the two subgroups were ascertained by the Mann-Whitney test. Statistical analyses were performed using the R (version 4.1.1), R base packages and R Bioconductor packages.

## 3 Results

### 3.1 Identification of Differentially Expressed Genes Between Normal and Tumor Tissues

The flow diagram was presented in Figure 1.103 FAM-related genes were obtained from GSEA website (gene set ID: GO_FATTY_ACID_CATABOLIC_PROCESS), and they were shown in Supplementary Table S1. In this study, the datasets of 502 tumor samples (including SCC of the oral cavity, larynx, oropharynx, hypopharynx, jaw, lip, and other sites of unclear origin) and 44 normal samples were from TCGA database. Based on our criteria of *p* < 0.05, a total of 69 DEGs were identified from 103 FAM-related genes (Figure 2).

**FIGURE 1:**
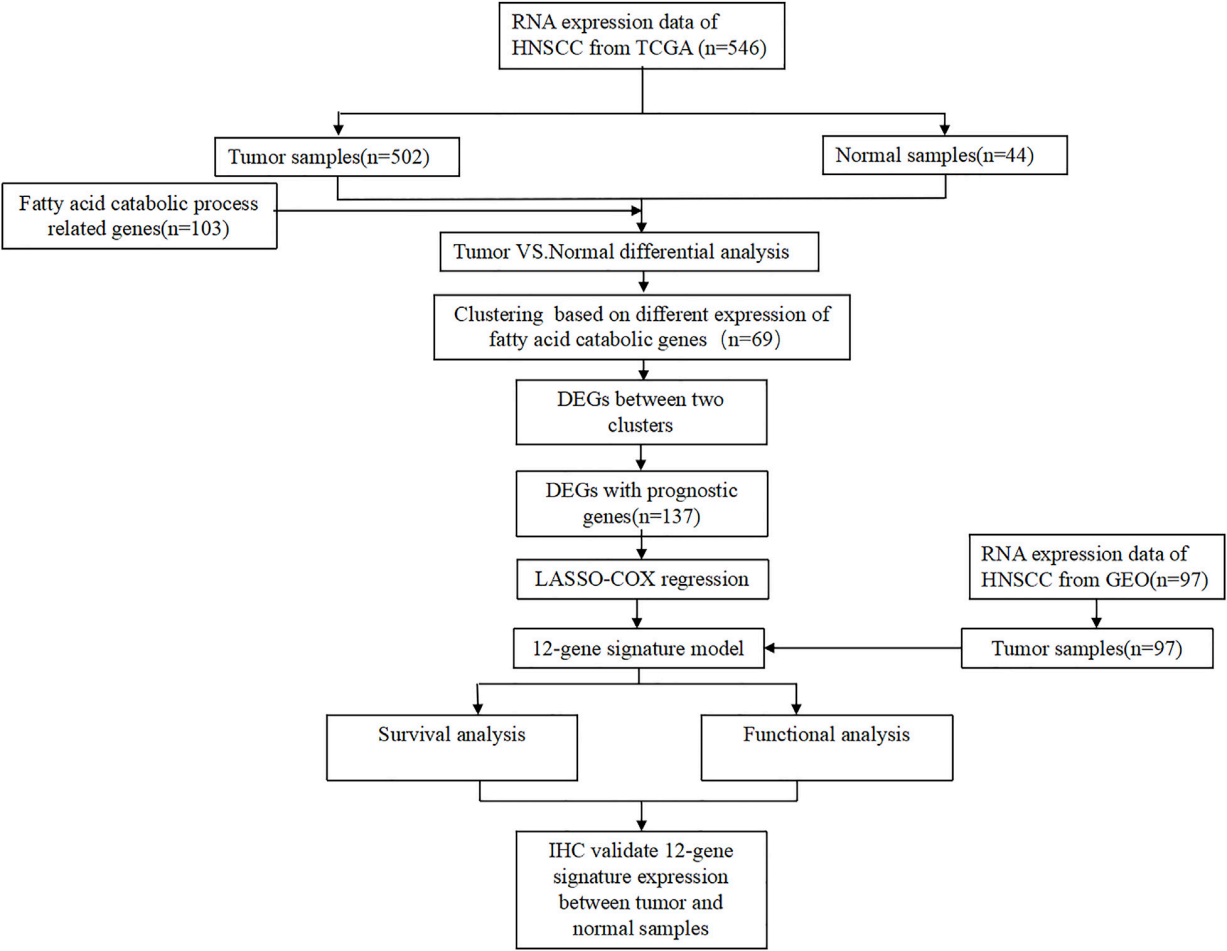
103 FAM-related genes were obtained from GSEA website.

**FIGURE 2:**
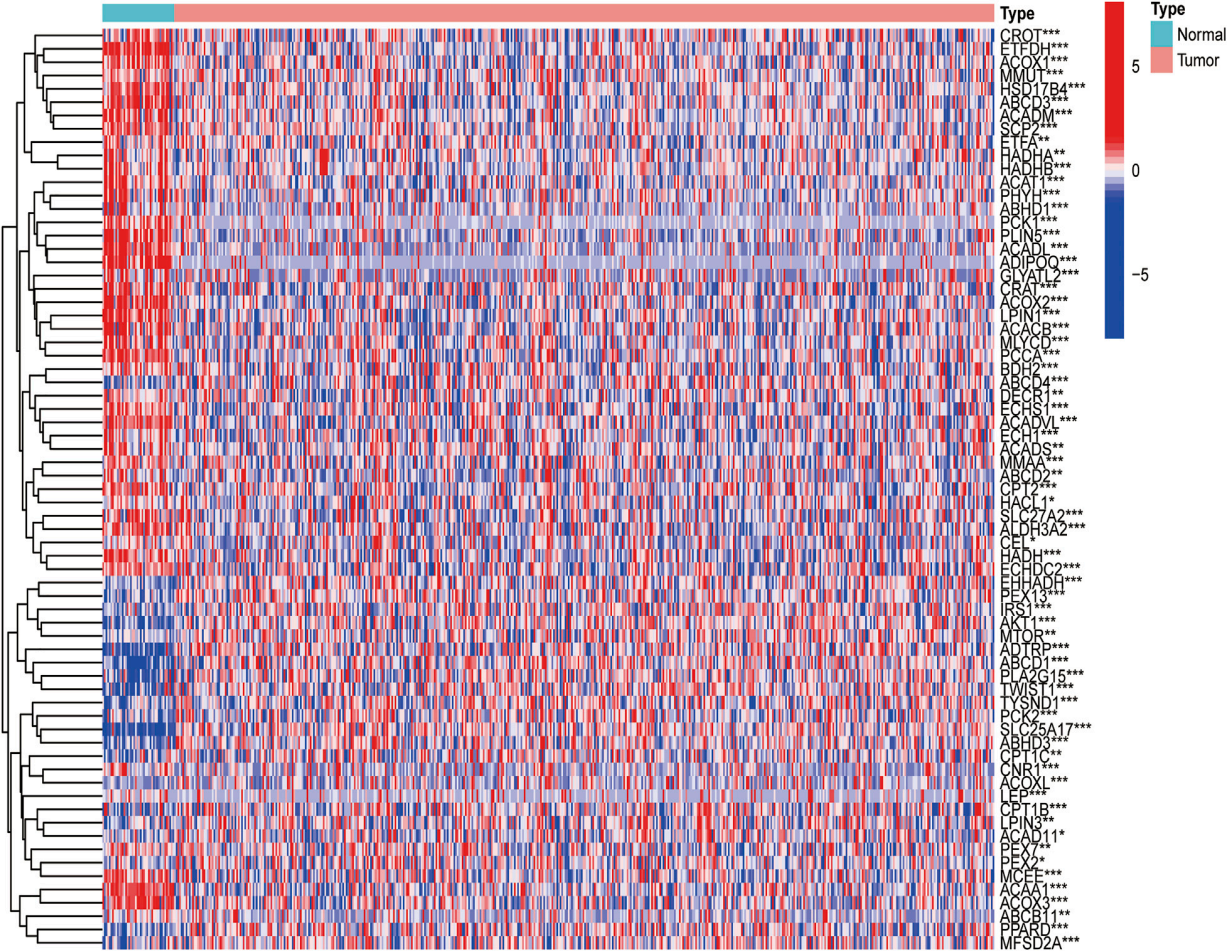
Expressions of the 33 FAM-related genes and the interactions among them. Heatmap (green: low expression level; red: high expression level) of the FAM-related genes between the normal (N, brilliant blue) and the tumour tissues (T, red). *p* values were showed as: ***p < 0.001; **p < 0.01; *p < 0.05.

### 3.2 Tumor Classification Based on the Fatty Acid Metabolism-Related Differentially Expressed Genes

**FIGURE 3:**
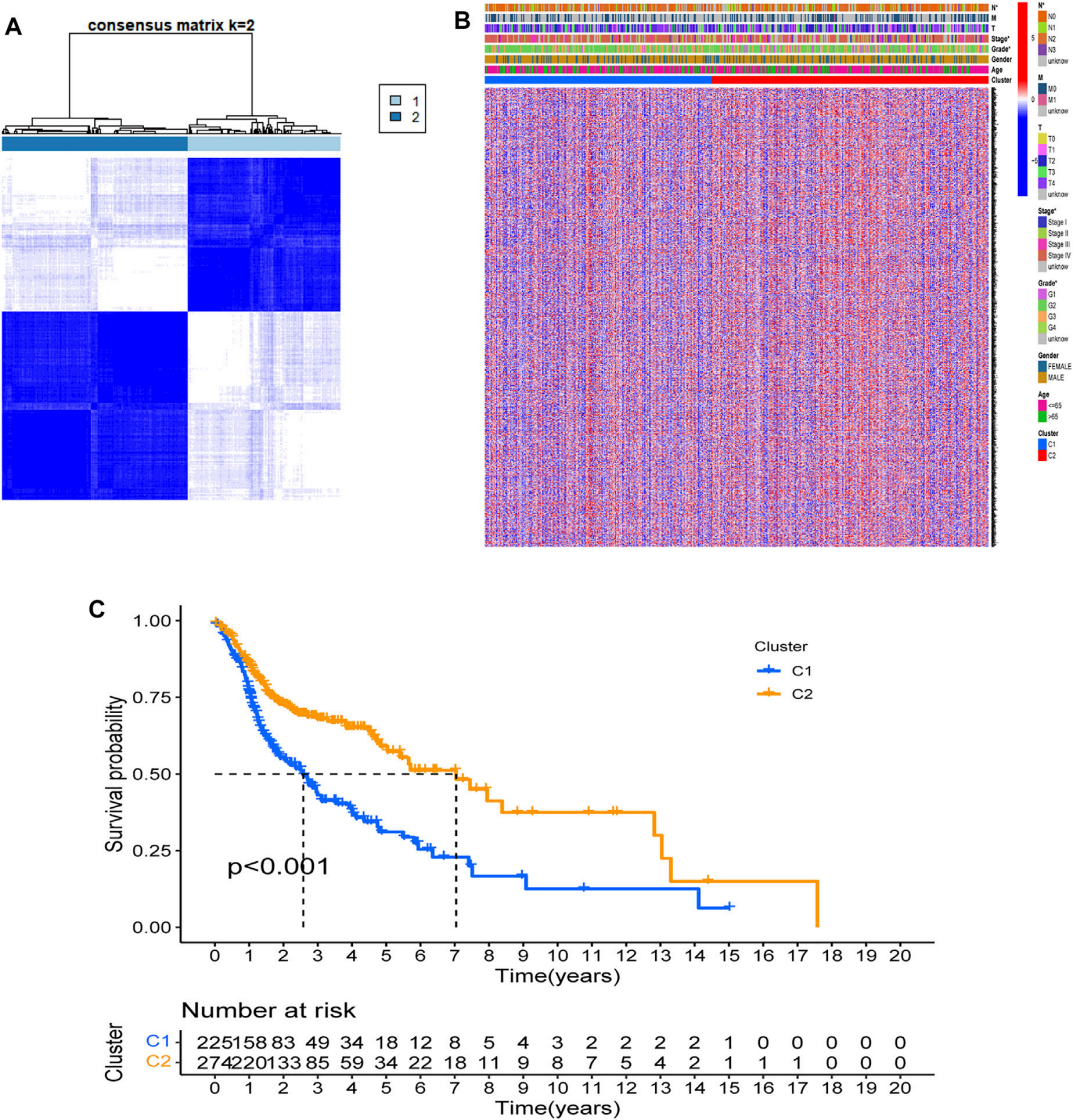
Tumor classification based on the FAM-related DEGs. **(A)** 546 HNSCC patients were grouped into two clusters according to the consensus clustering matrix (k = 2). **(B)**. Heatmap and the clinicopathologic characters of the two clusters classified by these DEGs. Two samples were more likely to be grouped into the same cluster when there was a higher consensus score between them in different iterations. **(C)**. Kaplan-Meier OS curves for the two clusters.

Based on expression data of 69 FAM-related DEGs, 546 HNSCC patients in the TCGA cohort were robustly separated into 2 subgroups via a consensus cluster analysis with the clustering variable (k) of 2 (Figure 3A). The gene expression profile of 69 FAM-related genes and the clinical features including the TNM stage, T stage, N stage, M stage, grade (G1-G4), gender, and age (>65 or ≤65 years) were presented in a heatmap, and there were significant differences across 2 subgroups with respect to TNM stage, N stage and grade (*p* < 0.05)​(Figure 3B). The overall survival (OS) was significantly different between the two clusters, the median survival values obtained by the Kaplan-Meier analysis is 2.57 years in cluster 1, while it is 7.04 years in cluster 2. There were differences in clinical phenotype, prognosis, and expression of FAM-related genes between Cluster 1 and Cluster 2. (*p* < 0.001, Figure 3C).

### 3.3 Construction of a Prognostic Model of Head and Neck Squamous Cell Carcinoma Based on Fatty Acid Metabolism-Related Genes

**FIGURE 4:**
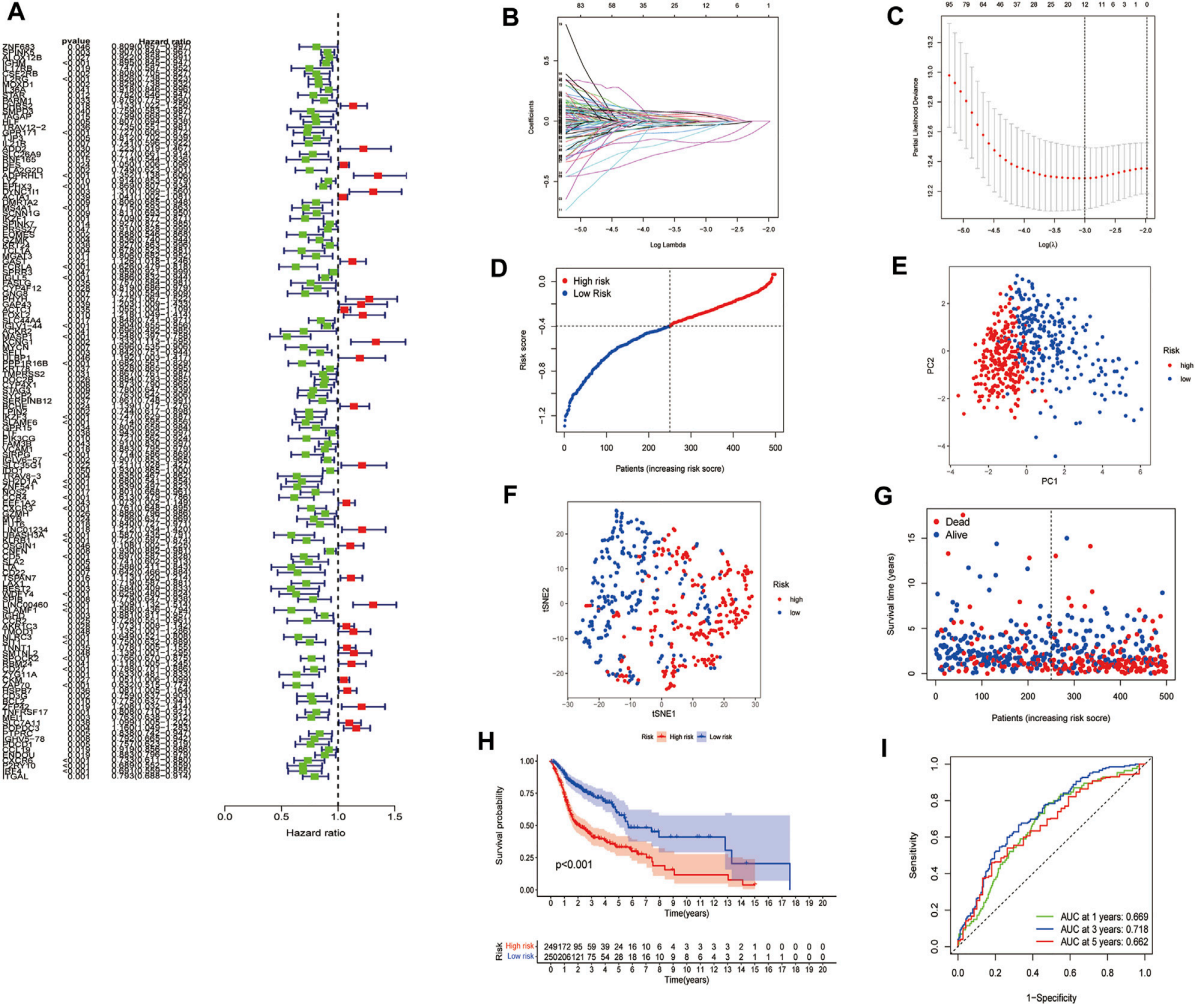
Construction of risk signature in the TCGA cohort. **(A)**. Univariate cox regression analysis of OS for each FAM-related gene. **(B)**. LASSO regression of the 12 OS-related genes. **(C)**. Cross-validation for tuning the parameter selection in the LASSO regression. **(D)**. Distribution of patients based on the risk score. **(E)**. PCA plot for HNSCC based on the risk score. **(F)**. t-SNE plot for HNSCC based on the risk score. **(G)**. The survival status for each patient. **(H)**. Kaplan-Meier curves for the OS of patients in the high- and low-risk groups. **(I)**. ROC curves demonstrated the predictive efficiency of the risk score.

First, a total of 546 HNSCC samples’ expression data from TCGA database and survival data were merged. Second, univariate Cox regression analysis showed that 137 FAM-related genes were related to the prognosis of HNSCC (*p* < 0.05). Among them, 30 genes were associated with increased risk with hazard ratios (HRs) > 1, while the other 131 genes were protective genes with HRs <1 (Figure 4A). Then, LASSO Cox regression analysis was conducted to identify the genes with the best prognostic value, and 12 optimal coefficients were obtained according to the optimum λ value at last (Figures 4B,C). The risk score was calculated based on the expression of the 12 genes and the risk score formula were as follows: $\mathrm{Risk}\;\mathrm{score}=\;\sum_{i=1}^{n}\mathrm{Coefi}*\mathrm{Expi}$ . 546 patients were divided into low- and high-risk subgroups by using the median score calculated by the risk score formula (Figure 4D). The PCA and t-SNE analyses further identified that HNSCC patients in different risk groups were well divided into two clusters (Figure 4E, Figure 4F). Patients in the high-risk group were more likely to have more deaths and a shorter survival time than those in the low-risk group (Figure 4G). The Kaplan-Meier survival analysis demonstrated notable survival discrepancies between the low- and high-risk subgroups (*p* < 0.001, Figure 4H). A ROC curve was applied to verify the sensitivity and specificity of the model. The areas under the curve (AUC) of the risk signature were 0.669 for 1-year, 0.718 for 2-years, and 0.662 for 5-years survival (Figure 4I).

### 3.4 Validation of the Risk Signatures

**FIGURE 5:**
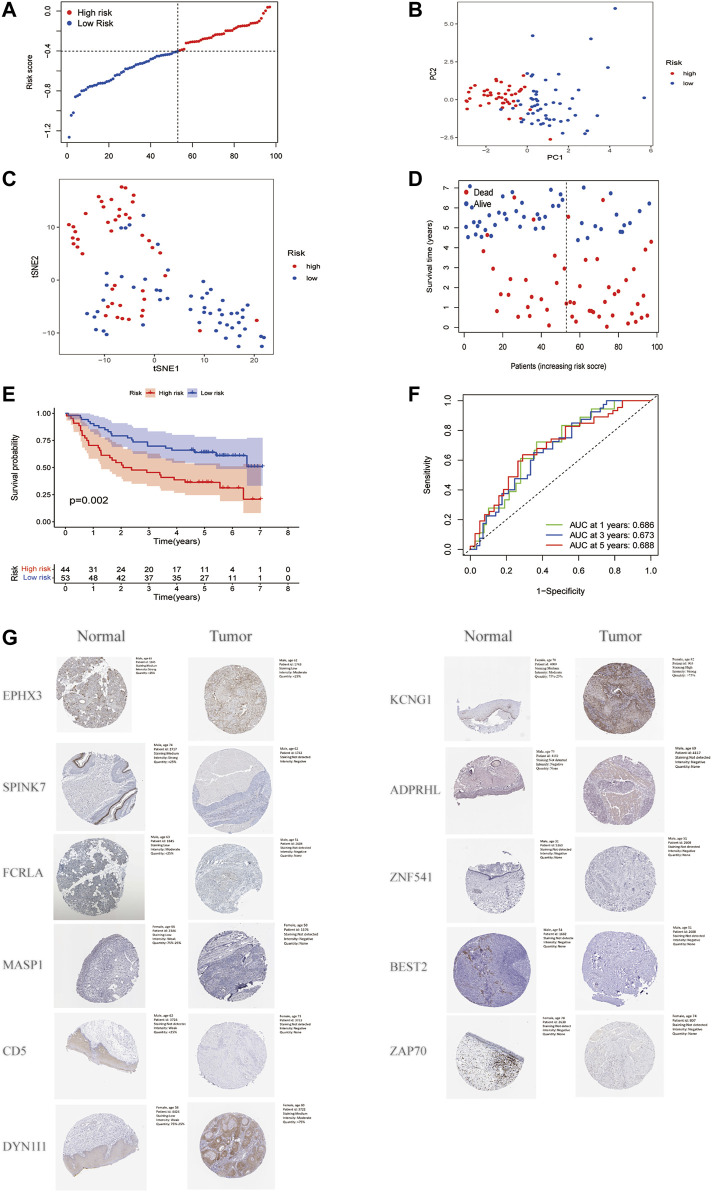
Validation of the risk model in the GEO cohort. **(A)**. Distribution of patients in the GEO cohort based on the median risk score in the TCGA cohort. **(B)**. PCA plot for HNSCC. **(C)**. t-SNE plot for HNSCC. **(D)**. The survival status for each patient. **(E)**. Kaplan-Meier curves for comparison of the OS between low-and high-risk groups. **(F)**. Time-dependent ROC curves for HNSCC. **(G)**. Verification of FAM-related gene expressions in normal and tumor tissue utilizing the Human Protein Atlas (HPA).

A total of 97 HNSCC patients from a GEO cohort were analyzed to verify the predictive performance of the risk model. Based on the median risk score in the training cohort, 97 patients in the GEO cohort were separated into the low-risk and high-risk subgroups (Figure 5A). The PCA and t-SNE analyses demonstrated significant separation between the two subgroups (Figure 5B, Figure 5C). Patients in the high-risk group were more likely to have more deaths and shorter survival time than those in the low-risk group. The median survival time was 2.02 years in the high-risk group and 5.04 years in the low-risk group (Figure 5D). Furthermore, the Kaplan-Meier analysis showed a significant difference in the OS between the low-and high-risk subgroups (*p* = 0.002, Figure 5E). In addition, ROC curve analysis of the GEO cohort revealed that the prognostic value of our model was high (AUC = 0.686 for 1-year, 0.673 for 2-years, and 0.688 for 5-years survival) (Figure 5F). EPHX3, SPINK7, FCRLA, MASP1, and CD5 had lower expression levels in HNSCC tissues, DYNC1I1 and KCNG1 had the higher expression levels by immunohistochemistry (IHC) analysis. The expression of ZNF541, BEST2, ADPRHL1, and ZAP70 were negative in both HNSCC tissues and normal tissues. The expression of LINC00460 in HNSCC tissues and normal tissues was not available (Figure 5G).

### 3.5 Independent Prognostic Value of the Risk Signature

**FIGURE 6:**
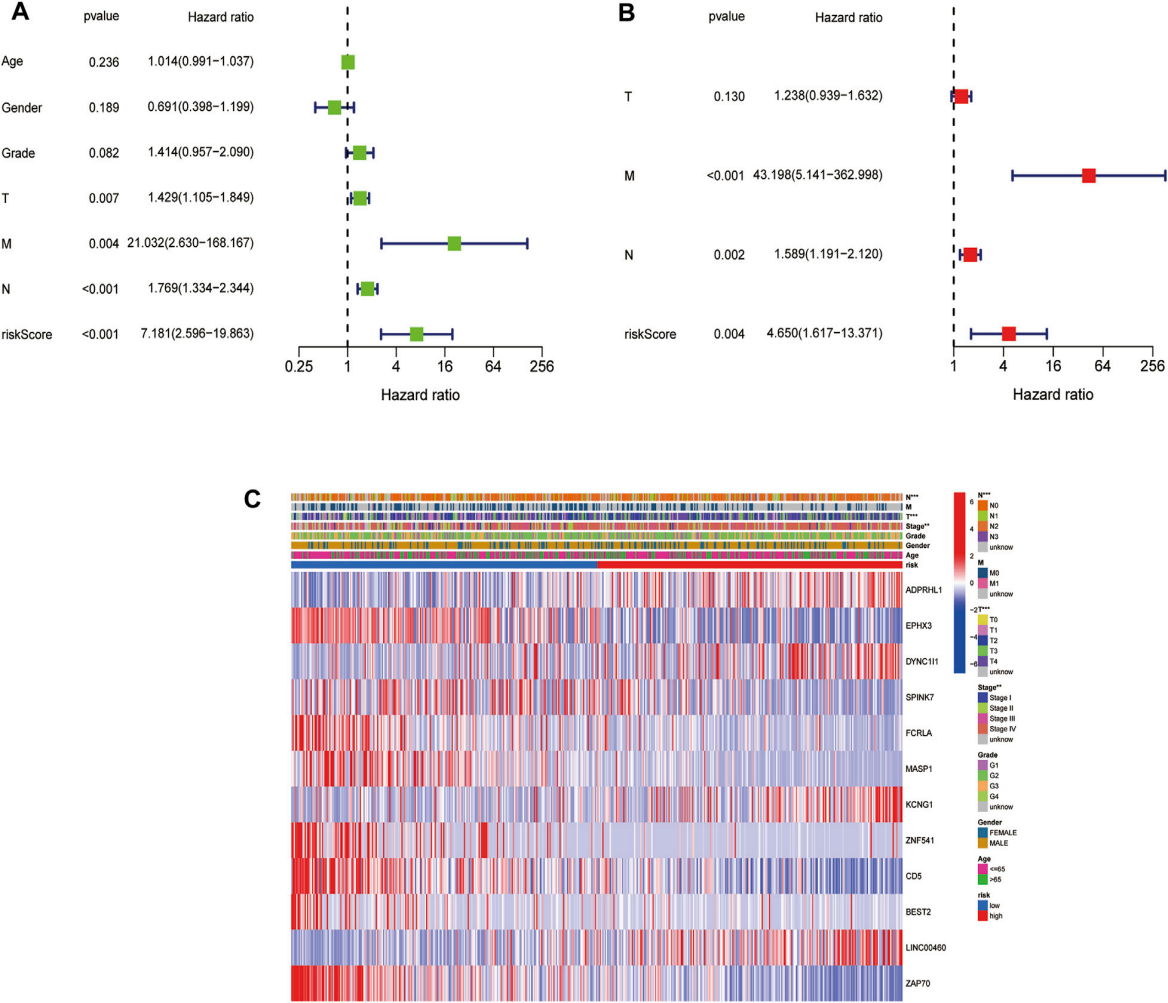
Univariate and multivariate Cox regression analyses for the risk score. **(A)**. Univariate analysis for the TCGA cohort. **(B)**. Multivariate analysis for the TCGA cohort. **(C)**. Heatmap (green: low expression; red: high expression) for the connections between clinicopathologic features and the risk groups (*p < 0.05).

The univariate Cox regression analysis indicated that the risk score was a prognostic factor for patients with HNSCC in the TCGA cohort (HR = 7.181, 95% CI: 2.596–19.863, Figure 6A). The multivariate analysis showed that the N stage, M stage, and the risk score (HR = 4.650, 95% CI: 1.617–13.371) were independent prognostic factors for patients with HNSCC in the TCGA cohort (*p* < 0.05, Figure 6B). Additionally, the TNM stage, T stage and N stage were found to be significantly different between the low- and high-risk subgroups (*p* < 0.05) (Figure 6C).

### 3.6 Functional Analyses Based on the Risk Model

**FIGURE 7:**
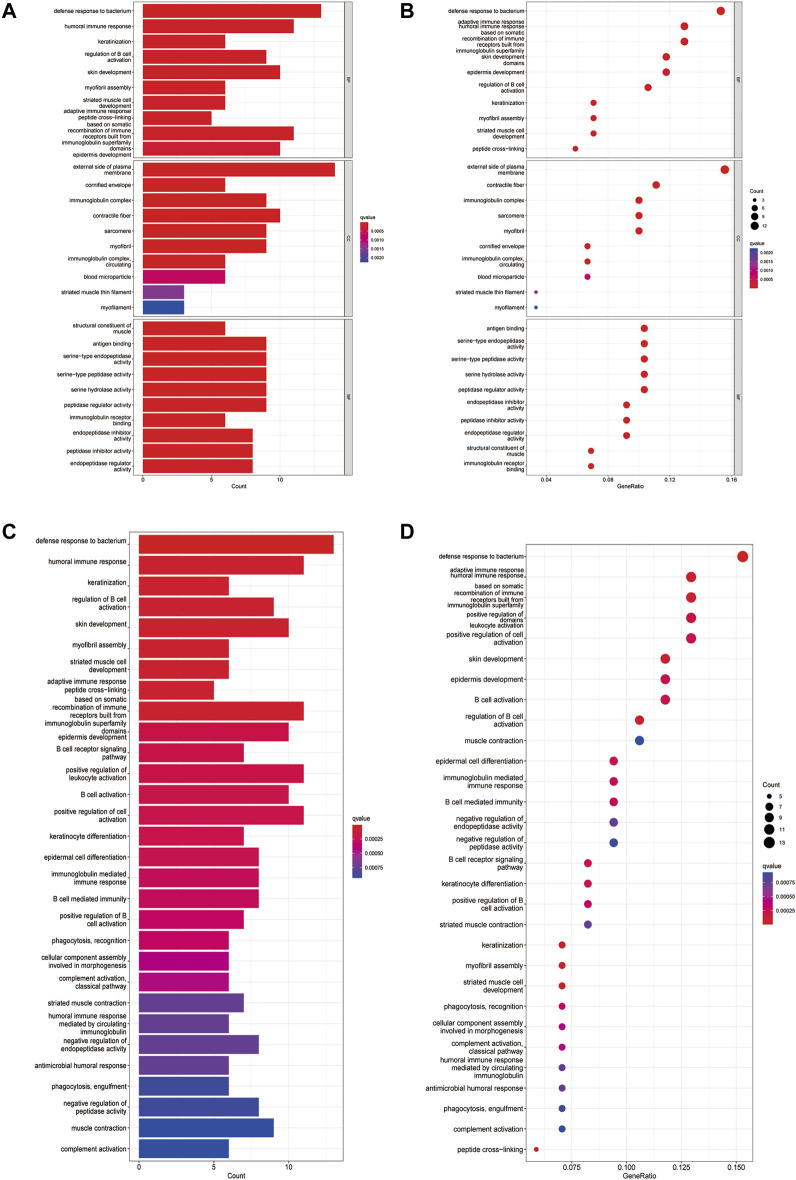
Functional analysis based on the DEGs between the two-risk groups in the TCGA cohort. **(A)**. Barplot graph for GO enrichment (the longer bar means the more genes enriched, and the increasing depth of red means the differences were more obvious). **(B)**. Bubble graph for GO enrichment (the bigger bubble means the more genes enriched, and the increasing depth of red means the differences were more obvious; q-value: the adjusted *p*-value). **(C)**. Barplot graph for KEGG pathways (the longer bar means the more genes enriched, and the increasing depth of red means the differences were more obvious). **(D)**. Bubble graph for KEGG pathways (the bigger bubble means the more genes enriched, and the increasing depth of red means the differences were more obvious; q-value: the adjusted *p*-value).

A total of 12 DEGs between the low- and high-risk subgroups in the TCGA cohort were found. Among them, 8 genes (FEPHX3, SPINK7, FCRLA, MASP1, ZNF541, CD5, BEST2 and ZAP70) were downregulated in the high-risk group, while the other 4 genes (ADPRHL1, DYNC1I1, KCNG1 and LINC00460)were upregulated. The results of GO functional enrichment analysis showed that these 12 DEGs were mainly enriched in the defense response to bacterium in the category of biological processes and the external side of plasma membrane in the category of cellular components. In terms of the category of molecular function, 12 DEGs were mainly enriched in the antigen binding, serine-type endopeptidase activity, serine-type peptidase activity, serine hydrolase activity, peptidase regulator activity and immunoglobulin receptor (Figure 7A, Figure 7B). KEGG enrichment analysis showed that hematopoietic cell lineage and cytokine-cytokine receptor interaction was the main pathway that DEGs involved, followed by Th1 and Th2 cell differentiation, Th17 cell differentiation, cell adhesion molecules and human T-cell leukemia virus infection (Figure 7C, Figure 7D).

### 3.7 Comparison of the Immune Activity Between High- and Low-Risk Subgroups

**FIGURE 8:**
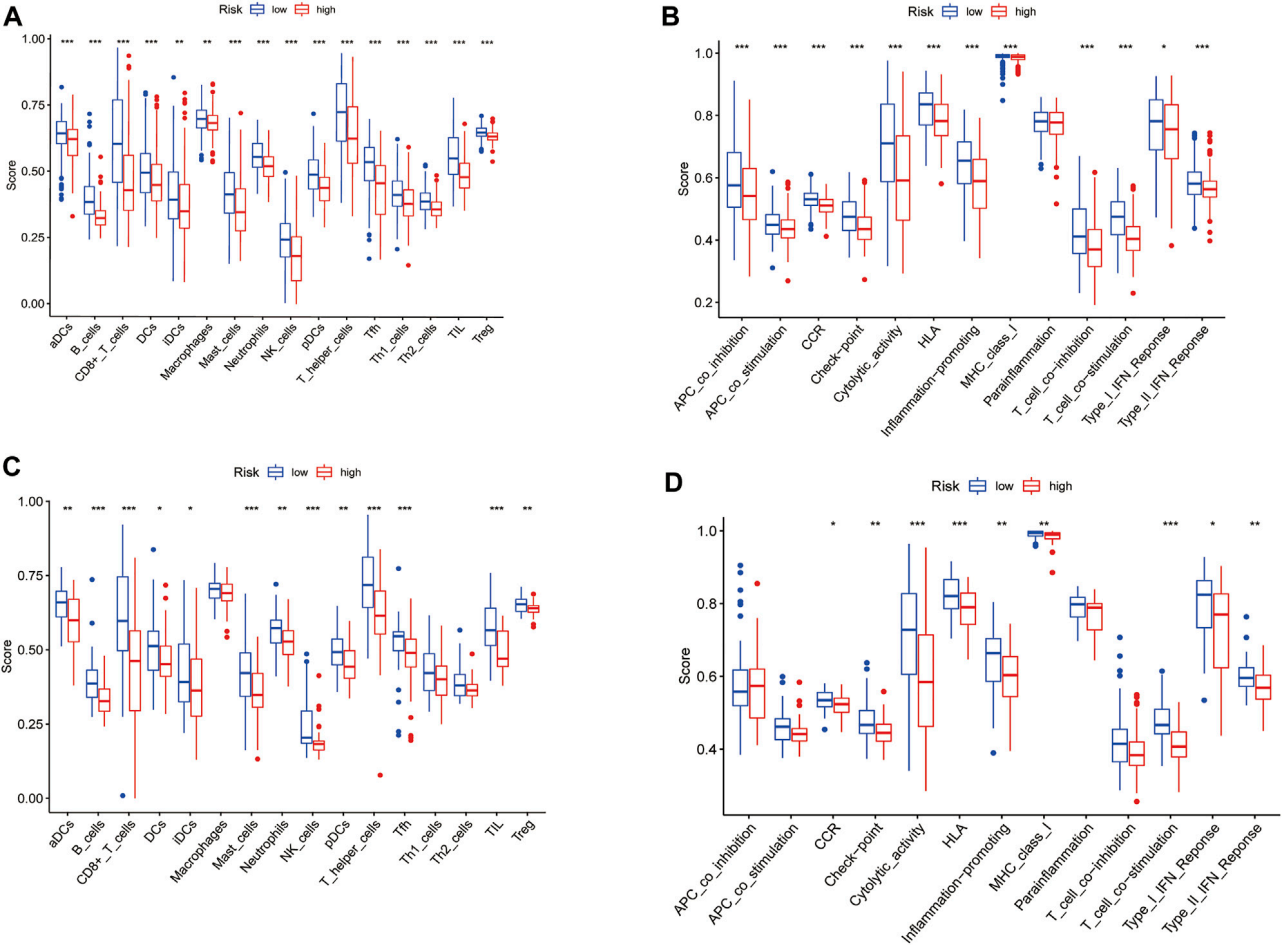
Comparison of the ssGSEA scores for immune cells and immune pathways. **(A,C)** Comparison of the enrichment scores of 16 types of immune cells and 13 immune-related pathways between low- (green box) and high-risk (red box) group in the TCGA cohort. **(B,D)** Comparison of the tumour immunity between low- (blue box) and high-risk (red box) group in the GEO cohort. *p* values were showed as: ns not significant; *p < 0.05; **p < 0.01; ***p < 0.001.

The enrichment scores of 16 types of immune cells and the activity of 13 immune-related pathways between the low- and high-risk groups in both the TCGA and GEO cohorts were evaluated by the ssGSEA method. 16 immune cells showed significantly higher levels of infiltration in the low-risk subgroup than the high-risk subgroup in the TCGA cohort, while 13 immune cells, including activated dendritic cells (aDCs), B cells, CD8^+^ T cells, dendritic cells (DCs), induced DCs (iDCs), mast cells, neutrophils, natural killer (NK) cells, plasmacytoid DCs (pDCs), T helper (Th) cells, tumor-infiltrating lymphocytes (TILs) and regulatory T (Treg) cells, were in the GEO cohorts (*p* < 0.05) (Figure 8A, Figure 8C). In the 13 immune pathways, except for the parainflammation and type-1 IFN response pathways, the remaining 11 pathways showed significantly higher activity in the low-risk group than in the high-risk group in the TCGA cohort (*p* < 0.001, Figure 8B). As for the GEO cohort, 9 out of 13 immune pathways illustrated higher activity in the low-risk group than in the high-risk group (*p* < 0.05). Among them, the cytolytic activity pathway, HLA pathway and T cell costimulation pathway showed significantly higher activity. (*p* < 0.001, Figure 8D).

## Discussion

In our research, we found 12 FAM-related genes related to the prognosis of HNSCC, and further constructed a prognostic risk model based on these genes to predict the prognosis of patients with HNSCC for the first time. Compared with single-gene biomarkers, the prognostic risk model composed of multiple genes was more accurate in predicting the prognosis of patients with HNSCC, thereby providing effective help for improving the therapeutic effect and prolonging survival of patients with HNSCC.

Studies have demonstrated that several FAM-related enzymes were increased in HNSCC compared with non-malignant tumors, but the relationship between FAM-related genes and the prognosis of HNSCC has remained unclear (Su et al., 2020). In the present study, using transcriptome data of 502 HNSCC samples and 44 normal samples released by TCGA database, we obtained a total of 69 DEGs FAM-related genes. The samples were divided into 2 clusters according to 69 DEGs via cluster analysis. Then DEGs in the two clusters were found, and 137 prognostic DEGs were identified by univariate analysis. Subsequently, combined with the clinical information of 546 HNSCC patients from TCGA database, a 12-gene prognostic risk model was established (FEPHX3, SPINK7, FCRLA, MASP1, ZNF541, CD5, BEST2 and ZAP70 were down-regulation, ADPRHL1, DYNC1I1, KCNG1 and LINC00460 were up-regulation) using multivariate Cox regression and LASSO regression analysis. The risk scores of 546 HNSCC samples were calculated. According to the median risk score, 546 HNSCC patients were divided into the high- and low-risk groups. The Kaplan-Meier survival analysis showed that the survival time of HNSCC patients was significantly shorter in the high-risk group than that in the low-risk group. The ROC curve (AUC value >0.60) verified the accuracy of the prognostic risk model. The same conclusion was obtained in the GEO dataset. After that, the multivariate Cox regression analysis indicated that the risk score was an independent factor for patients with HNSCC in the TCGA cohort. In addition, ssGSEA functional enrichment analysis was conducted to further investigate the relationship between the prognostic risk model and signaling pathways as well as immune status. The level of infiltrating immune cells was relatively low and the activity of immune-related pathways was decreased in the high-risk group compared with the low-risk group.

This study generated a signature featuring 12 FAM-related genes (ADPRHL1, DYNC1I1, KCNG1, LINC00460, EPHX3, SPINK7, FCRLA, MASP1, ZNF541, CD5, BEST2, ZAP70) and found that it could predict survival in patients with HNSCC. Among them, 8 of 12 hub genes (EPHX3, SPINK7, FCRLA, MASP1, ZNF541, CD5, BEST2, ZAP70) seemed to be cancer-promoting genes as they were downregulated in the high-risk group. Previous studies have suggested that the methylation of **EPHX3** (epoxide hydrolase 3), a metabolic enzyme that converted mutagenic epoxides into trans-dihydrodiols for detoxication, was considered as a predictive factor for the recurrence of prostate cancer, but as a favorable prognostic factor for HNSCC(Cottrell et al., 2007; Stott-Miller et al., 2014; Bai et al., 2019). The hypermethylation of EPHX3 was associated with salivary gland adenoid cystic carcinoma development and progression (Bell et al., 2011). High expression of EPHX3 increases ceramide linoleate epoxide hydrolysis and functions to control flux through the alternative and crucial route of metabolism by the dehydrogenation pathway of SDR9C7, which implicated a role for EPHX3 in tumor suppression (Edin et al., 2021). **SPINK7**, also named Kazal Type 7 or esophageal cancer-related gene 2 (ECRG2), was a type of protein from the family of serine peptidase inhibitor (Azouz et al., 2018). SPINK7 was identified as a tumor suppressor gene that could inhibit cell growth, suppress cell migration, invasion and metastasis, and promote cell apoptosis by inhibiting the binding of urokinase plasminogen-type activator (uPA) to uPA receptor (uPAR) and cleavage of uPAR(Huang et al., 2007; Cheng et al., 2010). A recent study has demonstrated that the expression of SPINK7 could be utilized to predict the molecular stage of the oral SCC lesions (Pennacchiotti et al., 2021). Another study showed that SPINK7 played an important role in skin homeostasis and inflammatory skin diseases (Chen H. et al., 2021). **FCRLA** (Fc receptor-like A) belongs to a family of Fc receptor like-molecules, and has been identified as a B cell-specific protein and may be involved in the development of lymphomas (Inozume et al., 2007; Fang et al., 2018). FCRLA has been confirmed to be related to the immune status and better prognosis of ovarian cancer (Fan et al., 2021). FCRLA was also found to be associated with the expression of CD19, CD20 and prognosis of laryngeal squamous cell carcinoma (Tagliabue et al., 2020). **MASP-1** (Mannose-binding lectin-associated serine protease 1) is a member of mannose-binding lectin-associated serine protease family and was first believed to up-regulate lectin pathway activation (Schwaner et al., 2017; Debreczeni et al., 2019). The role of MASP-1 in cervical cancer progression has been established (Maestri et al., 2018). However, MASP-1 was found to be a protective factor for HNSCC in our study. Similarly, another study reported that MASP-1 was found positively correlated with a better prognosis of hepatocellular carcinoma (Xu et al., 2021). **ZNF541** (The zinc finger protein 541) is located on chromosome 19 which possesses the highest gene density of all human chromosomes. Gene mutations on chromosome 19 were usually found to be associated with the occurrence of malignant tumors (Rose, 2018). Low expression levels of ZNF541 were related to the radiosensitivity of breast cancer (Yan et al., 2021). However, ZNF541 expression level was independently associated with a better OS of HPV-positive oropharyngeal cancer, which is similar to our study (Camuzi et al., 2021). **CD5**, a T-Cell surface glycoprotein, was mainly expressed on T cells and a small subset of normal B cells and was considered as an immunoregulatory biomarker in resectable non-small cell lung cancer and other cancer types (Moreno-Manuel et al., 2020). Future studies could investigate the association between CD5 and prognosis in HNSCC. **BEST2** (Bestrophin 2), a part of the bestrophin gene family of anion channels, is expressed predominantly in the retinal pigment epithelium and colon (Yu et al., 2010). It has been reported that high expression of BEST2 gene in HNSCC may lead to a good prognosis (Qin et al., 2020). To the best of our knowledge, the association of BEST2 gene and the prognosis of HNSCC was identified for the first time in our study. **ZAP70** (Zeta chain of T cell receptor associated protein kinase 70) is expressed in a broad range of B cell malignancies (Sadras et al., 2021). Chen et al. demonstrated that ZAP70 could promote cell survival, microenvironment interactions, protein synthesis and further drive disease progression in chronic lymphocytic leukemia cells (Chen et al., 2021). ZAP-70 could also shape the immune microenvironment in B cell malignancies (Chen et al., 2020). Liu et al. proposed that ZAP70-deficiency may improve reverse cholesterol transport and decrease the inflammatory response of T cells (Liu et al., 2019). Gong et al. pointed that an altered methylation pattern of ZAP70 is associated with poor survival of HNSCC (Gong et al., 2020).

The remaining 4 FAM-related genes (LINC00460, ADPRHL1, DYNC1I1, KCNG1) in the prognostic risk model were up-regulated in HNSCC tissues, suggesting that they may play a role in tumorigenesis and development of HNSCC. **LINC00460** (long intergenic non-protein coding RNA 460) is located on chromosome 13q33.2 and transcribed as a 913-nt transcript. Some studies showed that LINC00460 could promote tumor growth and malignant progression and is correlated with survival in multiple tumor types, including nasopharyngeal carcinoma, papillary thyroid carcinoma, esophageal cancer, gastric cancer, lung cancer, breast cancer, colorectal cancer, renal cell carcinoma, pancreatic cancer, bladder and urothelial carcinoma, ovarian cancer, and meningioma (Kong et al., 2018; Liu W. et al., 2018; Liu X. et al., 2018; Wang F. et al., 2018; Xing et al., 2018; Wen et al., 2019; Ye et al., 2019; Cisneros-Villanueva et al., 2021; Hou et al., 2021; Qian et al., 2021; Wu et al., 2021; Yang et al., 2021). HNSCC is certainly no exception, with its identification as a prognostic lncRNA signature using orthogonal partial least squares discriminant analysis (OPLS-DA) which integrates RNA-Seq data from TCGA database and matching clinical information from a large cohort of patients with HNSCC(Cao et al., 2017). LINC00460 is a promising candidate potential target for cancer therapy for HNSCC(Jiang et al., 2019). **ADPRHL1** (ADP-ribosylhydrolase like 1) is a member of the ADP-ribosylhydrolase protein family and a reversible posttranslational modification which could regulate protein function (Wan et al., 2018). Pilot studies have noted **DYNC1I1** (dynein cytoplasmic 1 intermediate chain 1) was an adverse factor for the prognosis of patients with liver hepatocellular carcinoma, gastric cancer, colon cancer and glioblastoma (Gong L.-B. et al., 2019; Gong L. et al., 2019; Sakthikumar et al., 2020; Yin et al., 2020; Zhou J. et al., 2021). There were no published researches on the ADPRHL1, DYNC1I1, KCNG1 in HNSCC before to our knowledge.

A previous study found that high abundances of CD3 or CD8 TILs were independently associated with prolonged survival outcomes among HNSCC patients (Kim et al., 2016). Multiple pieces of evidence showed a general increase of both circulating and infiltrating Treg during HNSCC development. Furthermore, Treg levels increased accordingly with tumor staging and they were particularly elevated in patients with active disease (Maggioni et al., 2017). Another retrospective study showed that increased intratumoral CD8^+^ T cell infiltration and an increased CD8^+^ T cell/Treg ratio were linked to a better treatment response of recurrent/metastatic HNSCC patients who were treated with immunotherapy (Hanna et al., 2018). The high infiltration of pDCs in tumors was also verified to promote the progression of HNSCC (Zhou B. et al., 2021). In our study, although CD8^+^ T cells and Treg cells were significantly higher in the low-risk group than those in the high-risk group, the number of Treg cells was lower than that in CD8^+^ T cells in either training set or test set. Therefore, CD8^+^ T cell/Treg ratio was better suited to predict the prognosis of patients with HNSCC. In addition, immune cell infiltration was significantly higher in the low-risk group than in the high-risk group in this study, which showed that the antigen presentation process, cellular immunity, and humoral immunity may be stronger in the low-risk group than in the high-risk group. It revealed that the risk prognostic model can partially reflect immune status in tumor microenvironment and has the potential to predict the efficacy of immunotherapy in patients with HNSCC.

There were still limitations in our study. Firstly, the prediction model was constructed based on the TCGA database and validated in the GEO database. But it has not been confirmed *in vivo* and *in vitro*. Secondly, only FAM-related genes were included in the prognostic model, thereby other important prognostic factors for HNSCC may have been excluded. This prognostic model deserves further in-depth studies.

## Conclusion

In summary, our study constructed a prognostic model of HNSCC based on 12 FAM-related genes for the first time. The model was proved to be an independent prognostic factor for survival in patients with HNSCC in both training set and test set, providing a new potential biomarker for prognosis prediction for patients with HNSCC. In addition, this prognostic model may be useful for evaluating immune status in tumor microenvironment of HNSCC, further screening the dominant patients with HNSCC of immunotherapy.

## Data Availability

The original contributions presented in the study are included in the article/Supplementary Material, further inquiries can be directed to the corresponding authors.

## Abbreviations

FAM, fatty acid metabolism; HNSCC, head and neck squamous cell carcinoma; TCGA, The Cancer Genome Atlas database; GEO, Gene Expression Omnibus; DEGs, differentially expressed genes; LASSO, least absolute shrinkage and selection operator; ROC, receiver operating characteristic; KEGG, Kyoto Encyclopedia of Genes and Genomes; GO, Gene Ontology; AUC, areas under the curve; PCA, Principal Component Analysis; OS, overall survival; t-SNE, t-distributed stochastic neighbor embedding; ssGSEA, single-sample gene set enrichment analysis.
